# Analysis of Resting-State fMRI Topological Graph Theory Properties in Methamphetamine Drug Users Applying Box-Counting Fractal Dimension

**DOI:** 10.18869/nirp.bcn.8.5.371

**Published:** 2017

**Authors:** Meysam Siyah Mansoory, Mohammad Ali Oghabian, Amir Homayoun Jafari, Alireza Shahbabaie

**Affiliations:** 1. Department of Medical Physics & Biomedical Engineering, School of Medicine, Tehran University of Medical Sciences, Tehran, Iran.; 2. Department of Neuro-Imaging and Analysis, Research Center for Molecular and Cellular Imaging, Tehran University of Medical Sciences, Tehran, Iran.; 3. Research Center for Biomedical Technologies and Robotics, Tehran University of Medical Sciences, Tehran, Iran.; 4. Iranian National Center for Addiction Studies, Tehran University of Medical Sciences, Tehran, Iran.; 5. Substance Abuse and Dependence Research Center, University of Social Welfare and Rehabilitation Sciences, Tehran, Iran.

**Keywords:** Graph theory, Box-counting fractal dimension, Mutual information, Linear correlation, Methamphetamine

## Abstract

**Introduction:**

Graph theoretical analysis of functional Magnetic Resonance Imaging (fMRI) data has provided new measures of mapping human brain in vivo. Of all methods to measure the functional connectivity between regions, Linear Correlation (LC) calculation of activity time series of the brain regions as a linear measure is considered the most ubiquitous one. The strength of the dependence obligatory for graph construction and analysis is consistently underestimated by LC, because not all the bivariate distributions, but only the marginals are Gaussian. In a number of studies, Mutual Information (MI) has been employed, as a similarity measure between each two time series of the brain regions, a pure nonlinear measure. Owing to the complex fractal organization of the brain indicating self-similarity, more information on the brain can be revealed by fMRI Fractal Dimension (FD) analysis.

**Methods:**

In the present paper, Box-Counting Fractal Dimension (BCFD) is introduced for graph theoretical analysis of fMRI data in 17 methamphetamine drug users and 18 normal controls. Then, BCFD performance was evaluated compared to those of LC and MI methods. Moreover, the global topological graph properties of the brain networks inclusive of global efficiency, clustering coefficient and characteristic path length in addict subjects were investigated too.

**Results:**

Compared to normal subjects by using statistical tests (P<0.05), topological graph properties were postulated to be disrupted significantly during the resting-state fMRI.

**Conclusion:**

Based on the results, analyzing the graph topological properties (representing the brain networks) based on BCFD is a more reliable method than LC and MI.

## 1. Introduction

Among the most intricate networks in nature is the human brain, which transports signals between specific brain regions and responds to external stimuli. The study of brain connectivity, therefore, depends to a great extent, on the comprehension of brain functions and pathology (Sporns & Zwi, 2004).

Functional Magnetic Resonance Imaging (fMRI) is an imaging technique applied to study human brain function and neurological diseases (Smith, Matthews, & Jezzard, 2001). Blood Oxygenation Level Dependent (BOLD) contrast, on the basis of varied magnetic properties of oxygenated (diamagnetic) and deoxygenated (paramagnetic) blood, is applied by well-liked techniques in fMRI (Fransson, 2005). fMRI benefits over the other functional imaging modalities, namely Electro Encephalography (EEG), Magneto Encephalography (MEG) and Positron Emission Tomography (PET) comprise its noninvasiveness, better spatial resolution compared to other modalities and short-time image attainment (Culham & Kanwisher, 2001).

In literature, the term “network” has various definitions. In graph theory and complex networks, “network” bluntly implies a set of nodes and pair-wise edges, by which the nodes are connected. This sense is referred to as Graph theoretical analysis of brain networks. “Network”, in neuroimaging, may designate a group of voxels or Regions of Interest (ROIs), that at resting state or in specific cognitive tasks, act identically (Sun, Tong, & Yang, 2012).

From the standpoint of complex networks, there has been a growing interest, over the recent years, in studying the wide-ranging brain activity interaction structure with the use of graph theory and centered on fMRI in the areas of addiction (Sutherland, McHugh, Pariyadath, & Stein, 2012), schizophrenia (Cabral, Kringelbach, & Deco, 2012), brain injury (Nakamura, Hillary, & Biswal, 2009), neuralgia (Zhang et al., 2014), epilepsy (Ponten, Bartolomei, & Stam, 2007), Alzheimer (Supekar, Menon, Rubin, Musen, & Greicius, 2008) and the like (Bullmore & Sporns, 2009).

A brain network, in graph theoretical analyses of fMRI data, is taken into account as an undirected graph, G=(V, E), where a node/vertex (V) in the graph delineates a brain region (i.e. ROI) and an edge/link (E) between two nodes is indicative of brain regions being functionally connected (Deuker et al., 2009).

To create a graph, the concept and also the definition of the edge is a challenging stage. The edge definition methods and the importance of each is well evaluated and tested in Smith study (Smith et al., 2011).

However, to assess the interaction strength between two brain regions for the edge definition, the LC (Pearson) coefficient of corresponding time series is most commonly utilized in functional connectivity studies, substantially in addiction research studies (Xia & He, 2011). The drawback of such a choice may be that the linear correlation does not take into account the nonlinear dependences possibly occurring in the data. While linear measures, including the Pearson correlation coefficient or coherence are frequently applied, increased attention is being paid to potential benefits of nonlinear measures (Donges, Zou, Marwan, & Kurths, 2009; Kreuz et al., 2007). Mutual Information (MI) can establish a nonlinear relationship between fMRI time series and provides an effective pure nonlinear noise-robust correlation measure (Bassett & Bullmore, 2009).

Research studies contemplating nonlinearity as an inherent feature of the brain dynamics are getting continuously more interested in nonlinear approaches toward the analysis of the brain signals, especially those measures in accord with the analysis of chaotic non-linear dynamical systems to analyze the resting state fMRI data, signifying that the presupposition of linearity might be oversimplifying (Hlinka, Paluš, Vejmelka, Mantini, & Corbetta, 2011). On the other hand, the brain, with fractal structure complexity, is best modeled as a complex system (Papo, Zanin, & Buldú, 2014). For the complexity of the brain signal, we can also assess the resting BOLD fMRI time series (Warsi, 2012). The fMRI time series within any given voxel, rather than correlating the brain areas connectivity with the use of measures of linearity and nonlinearity, can reveal the resting state brain functional network complexity. By complexity we mean the spatial distribution of fMRI signals phase that should not be confused with brain complexity.

The most frequently applied method for analyzing the physiological signals complexity is fractal analysis (Ahmadi, Ahmadlou, Rezazade, Azad-Marzabadi, & Sajedi, 2013). Fractal Dimension (FD) analysis is likely to include new information on the functional connectivity of the brain (Sporns, 2006). This information is not attained by applying any traditional linear, as well as nonlinear measures. However, it should be noted that selection of the appropriate method to determine the graph edges in functional connectivity studies and the impact of each edge-determination method on the network topological features is not fully determined yet (Papo et al., 2014).

In this study, the main hypothesis is that the features extracted from the graph theory-based brain network in methamphetamine abusers, compared to healthy individuals, are subjected to modifications. Another hypothesis investigated in this study was that among the edge-definition criteria (LC, MI and BCFD) which will have a better performance, regardless of the connectivity threshold, to differentiate the topological features extracted from the brain networks of the addicts and the control group.

## 2. Methods

**Figure 1.**
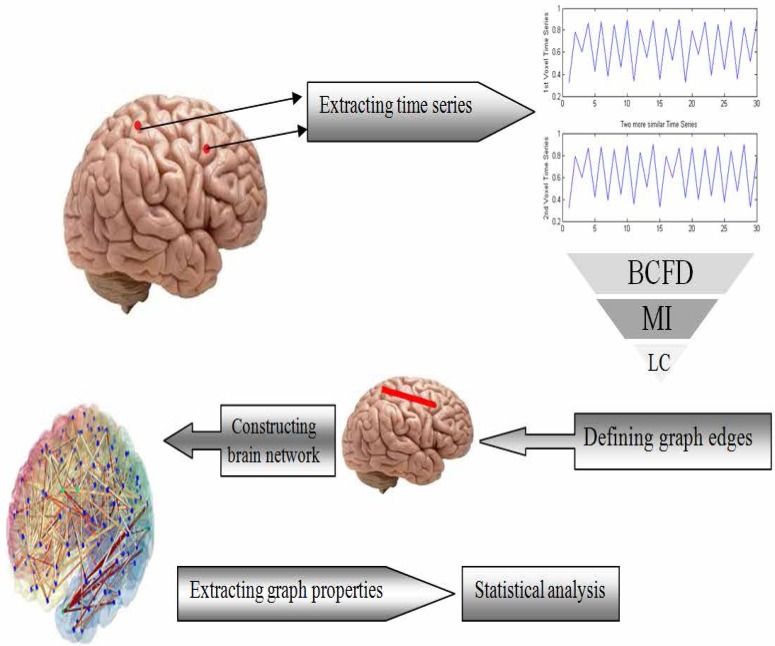
The flowchart of the proposed methods.

The flowchart of the proposed method, including data acquisition, data preprocessing, functional graph construction, topological graph properties and statistics is depicted in Figure 1. As shown in it, in the first phase, fMRI data are acquired and processed and time series are extracted. Then, using LC, MI and BCFD, the edges are defined and the brain network is constructed. Finally, statistical analysis is performed. Each phase is distinctly stipulated in the following sections.

### 2.1. Subjects

A total of 18 normal controls (NCs: 18, aged 23–46 years, mean±SD=31.67±7.98 years, right-handed) were enlisted from the local community. Additionally, we enrolled 17 age-matched methamphetamine-dependent individuals (MDIs: 17 Males, aged 22–39 years, mean±SD=30.52±4.57 years, right-handed). Demographic and clinical information regarding MDIs is demonstrated in Table 1. Mann-Whitney U test (Bergfeldt, Jonsson, Bergfeldt, & Julin, 2015) showed no significant difference between MDIs and NCs (P=0.85).

**Table 1.** Demographic characteristic of MDIs.

**Descriptive Statistics**	
Gender (men)	17/17
Age, y	30.52±4.57
Education, y	12±2.91
Duration of methamphetamine abstinence, d	13.31±4.64
Duration of addiction, y	3.50±1.74
Number of subjects with history of opium abuse	11/17
Number of subjects with history of heroin abuse	8/17
Number of subjects with history of crystalline heroin abuse	9/17
Number of subjects with history of alcohol abuse	12/17
Number of subjects with history of hashish abuse	12/17
Number of subjects with history of cocaine abuse	4/17
Number of subjects with history of cigarette smoking	12/17

All subjects were capable to realize and follow the protocol of the study during imaging. Neither MDIs nor NCs had any history of pain, neurologic or psychiatric disorders, head injuries, schizophrenia or an affective disorder, in line with their medical history. The present paper was performed in agreement with the Helsinki Declaration also, confirmed by the Research Ethics Review Board of Tehran University of Medical Sciences in Tehran, Iran. Prior to the MRI scanning, informed written consent was obtained from each subject.

### 2.2. Data acquisition

On a 3 Tesla Siemens Tim Trio scanner, MRI data were acquired in Medical Imaging Department at Imam Khomeini Hospital, Tehran, Iran. To acquire data for Resting-State Magnetic Resonance Imaging (RS-fMRI), a T2*-weighted gradient-echo Echo-Planar Imaging (EPI) sequence was used with the following parameters: Time of Repetition (TR)=3000 ms, Time of Echo (TE)=30 ms, flip angle=90°, matrix=64×64, field of view (FOV)=192 mm^2^, thickness/gap=4.5 mm, 22 axial slices covering the whole brain and 240 volumes attained in almost 8 minutes. Besides, via a T1-weighted 3D turbo-gradient-echo sequence (TR=1800 ms, TE=30 ms, flip angle=90°, matrix=256×256, FOV=230×230 mm^2^, thickness=1.0 mm, and 160 sagittal slices), brain structural images with 3D high resolution were obtained. The participants were supposed to have their eyes closed, keep calm, not to systematically think about anything, and not to fall asleep. None of the lights in the scanner room were on during the RS-fMRI scanning.

### 2.3. Data preprocessing

All the fMRI data were preprocessed using MATLAB software and DPARSF_V2.0 Toolbox (Chao-Gan & Yu-Feng, 2010). For each individual, in the first place, the first 10 volume images were excluded from the RS-fMRI data to make the subjects adapt to the environment and to stabilize the scanner, letting 260 volumes remain for supplementary analysis. Then, to correct the acquisition time delay between slices within the similar TR, slice timing was administered. After that, for the correction of the inter-TR head motions, realignment to the first volume was done. Next, we spatially normalized the fMRI data to a standard Montreal Neurological Institute (MNI) (Tzourio-Mazoyer et al., 2002) template and implemented resampling to a voxel size of 36×63 mm^3^. According to the studies conducted so far, no spatial smoothing was applied (Achard & Bullmore, 2007; Braun et al., 2012; Wang et al., 2009). Eventually, to reduce the low-frequency drift and high-frequency physiological noise, band-pass filtering was performed for each voxel at the frequency of 0.01–0.08 Hz. The RS-fMRI data for each subject were checked for head motion. In accord with the criteria that the translation and rotation of head motion in any direction were not more than 1.5 mm or 1.5, none of the subjects were disqualified.

### 2.4. Graph construction

Topological properties of the brain were investigated through the method of binary graph G=(V, E), where a brain region (i.e. ROI) and an edge (E) between two nodes in the graph are indicated by a node/vertex (V). All the regarded graphs in this work are undirected. To analyze complex network, we applied a simple generalization of the graph called weighted graph (Bollobás, 1998).

In this paper, based on the previously conducted studies, two common methods (i.e. LC (Hlinka et al., 2011) and MI (Hartman, Hlinka, Paluš, Mantini, & Corbetta, 2011)), as well as the proposed method (i.e. BCFD) were applied for edge definition. For graph construction, node and edge need to be defined, each of which is explained in detail as follows:

#### 2.4.1. Node definition

In order to have the brain parcellated into 90 Regions Of Interest (ROIs) (45 in each hemisphere), an Automated Anatomical Labeling (AAL) atlas (Tzourio-Mazoyer et al., 2002) was utilized to construct the brain functional networks for each participant.

#### 2.4.2. Edge definition

At this stage, the connectivity, dependence and interactions between the brain regions, i.e. the graph nodes, have to be quantified. Edge definition phase to create graph functional connectivity is challenging to some extent, for which various methods, including linear and nonlinear are presented so far (Ahmadlou & Adeli, 2011; Ahmadlou, Ahmadi, Rezazade, & Azad-Marzabadi, 2013). It should be noted that the method considered as the best method for edge definition and as the edge definition gold standard is still a questionable issue (Papo et al., 2014). According to the previous studies, we used LC and MI methods as the representatives of the linear and nonlinear methods, respectively, together with the proposed method “BCFD” for quantifying the interactions between 90 regions selected as nodes based on AAL Atlas. Then, BCFD method was evaluated in comparison with LC and MI. To quantify the nodes connectivity, each of these three methods considers a different criterion.

LC method quantifies the interactions between the time series of each node with linearity supposition, while MI lacks the assumption of the linearity between the brain regions and proposes an index for the evaluation of the shared information between the two time series. But this method is sensitive to the length of time series being analyzed. Since, in functional connectivity studies, the length of time series is usually short, MI use can be somewhat challenging. BCFD was applied in the current study, as it is based on the concept of self-similarity and complexity, i.e. distribution of the points in the phase space, calculating the connectivity and interactions of the brain regions. In addition, it does not assume interactions linearity or sensitive to the length of the time series. The concepts of “self-similarity” and “complexity” have not been evaluated using the graph theory in the functional connectivity studies. We computed the time series for each ROI, by averaging the signals of all voxels within that region. By calculating the Pearson correlation coefficient, MI and BCFD in the residual time courses between all ROI-pairs, a 90×90 adjacency matrix was attained for each subject.

##### 2.4.2.1. Pearson correlation coefficient

A value for the linear association between the time series to be quantified is Pearson correlation, by which the dependence structure Gaussianity presupposition is given. Regarding {X}={x_1_,x_2_,x_ND_} and {Y}={y_1_,y_2_,y_ND_} as the voxel’s time series, the number of components in each set would be. You will get the Pearson correlation coefficient r as the following equation (Gómez, Vaquero, López-Mendoza, González-Rosa, & Vázquez-Marrufo, 2004) in which is the mean of X and is the mean of Y.

(1) $$r=\frac{\sum_{i=1}^{N_{D}}(x_{i}-\bar{x})(y_{i}-\bar{y})}{{\left\{\sum_{i=1}^{N_{D}}{\left(x_{i}-\bar{x}\right)}^{2}\right\}}^{\frac{1}{2}}{\left\{\sum_{i=1}^{N_{D}}{\left(y_{i}-\bar{y}\right)}^{2}\right\}}^{\frac{1}{2}}}$$

##### 2.4.2.2. Mutual Information (MI)

An effectual noise-robust correlation value and a non-random association between time series can be developed and constructed by mutual information (Khan et al., 2007). To predict MI, we can predict the average number of bits of one of the two time series by expressing the value of the other. Therefore, quantifying the variable X, the average number of the variable Y (i.e. mutual information of the variables X and Y, represented by I(X, Y)) can be predicted by measuring variable X. As a result, we will have MI as (Bonita et al., 2014; Ward & Mazaheri, 2008):

(2) $$I(X,Y)=\sum_{i=1}^{N_{X}}\sum_{i=1}^{N_{Y}}P{\left(i,j\right)}_{XY}\log_{2}\left\{\frac{P{\left(i,j\right)}_{XY}}{P{\left(i\right)}_{X}P{\left(j\right)}_{Y}}\right\}$$,

and correspondingly designate the number of bins in the variable X’s histogram and the number of components in the variable Y’s histogram, in which does not vitally equal, in general. Besides, the probability that a component in the i^th^ bin of the X axis segregation and the j^th^ bin of the Y axis segregation would respectively constitute an (x, y) pair is the joint probability distribution, i.e. P_XY (i,j)_.

##### 2.4.2.3. Box-Counting Fractal Dimension (BCFD)

In order to predict fractal dimension, we employ self-similarity being conceptualized as the fundamental principle. The equation below is given for the FD of a bounded set A:

(3) D=lim⁡r→0log⁡(Nr)log⁡(1r)

**Figure 2.**
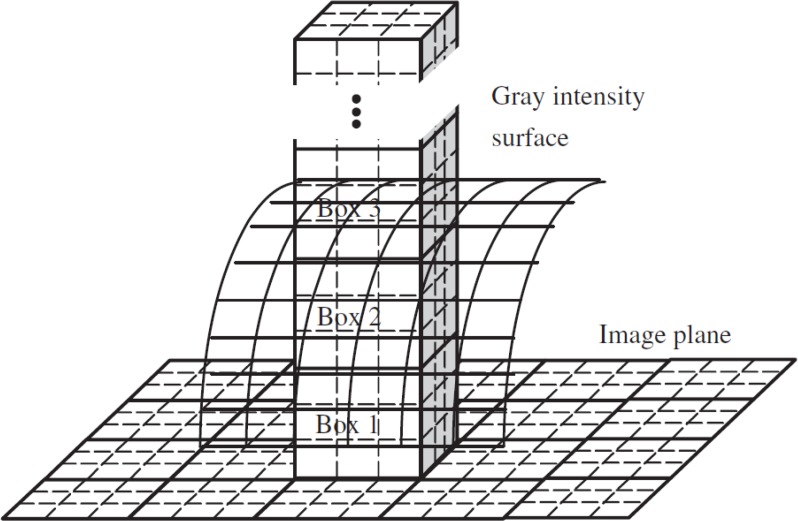
Sketch of determination of the number of boxes by the DBC method (Li et al., 2009).

In this regard, the minimum number of A’s discrete copies in the scale r is designated as N_r_. We can compute FD just for fractals that are deterministic. Via a box-counting method, a D estimate (i.e. the box-counting DB) can be computed. In the following, we explain the DBC method (Li, Du, & Sun, 2009). In this case, by plotting each time series of fMRI voxel’s against the other, we are able to create an image of the size M×M (M equals the length of fMRI time series). This image is regarded as a 3D spatial surface, in which the position of pixel on the plane of the image and the gray level of pixel are denoted by (x,y) and (z), the third coordinate. The plane, in this method, is partitioned by the s×s blocks. In each block’s scale (i.e. r=s), s represents an integer and 1<s≤M/2. In Figure 2, a column of s×s×ś boxes can be observed. Regarding G as the whole number of gray levels, G over equal to M over s. In addition, each box’s height is displayed by ś. As depicted in Figure 2, numbers 1, 2, 3, 4 … can be devoted to the boxes. The formula for the calculation of the number of boxes (k^th^ and l^th^), by which the (i,j)^th^ block is covered, would be as follows:

(4) (i,y)=l−k+1

Additionally, having taken all blocks’ contribution into consideration, is given for the varied measures by:

(5) $${\text{N}}_{\text{r}}=\sum_{\text{i},\text{j}}\text{n}(\text{i},\text{j})\text{r}$$

Ultimately, the minimum squares linear fit of log (N_r_) versus log (l/r) would result in FD being prognosticated.

### 2.5. Surrogate data

By generating the surrogate data, we aim at evaluating the performance of each method in several pairs of time series, before their applying in the real data. That is because these methods are used as the similarity measure of two time series from two fMRI voxels. We applied the outlined ideas, comparing the total MI, LC and BCFD between the signals in surrogate datasets. These surrogates are generated using the logistic equation (Arora & Santhanam, 2014) and randomization process.

This approach provides us with the opportunity to both test and quantify the deviation from linearity, providing a principled guide in judging the appropriateness of LC, MI and BCFD as measures of FC.

(6) x(n+1)=A.x(n).(1−x(n))

We exploited logistic equation and increased the A value randomly between 3<A<4. With an increase in the value of A within 3<A<4, we will be able to create time series with artificially increasing complexity and evaluate graph edge definition methods as a criterion for the connectivity between the brain regions in the presence of complexity. In the present study, complexity means the extent of the phase space i.e. the two series that have less phase space extent have more similarity and connectivity and vice versa. Therefore, increased complexity is interpreted as the similarity and connectivity decrease in the two time series. Hence, in logistic equation, values close to 3 mean more similarity and less complexity of the time series and vice versa.

In order to better assess the similarity measures applied in the detection of brain functional connectivity using the graph theory, other surrogate data were generated using the randomization process. In these data, at first two time series were randomly generated. Then, the similarity of these two time series to each other was gradually reduced to a specific amount. In the last phase, similarity measure value on each of these signal pairs by three proposed methods, including linear correlation, mutual information and fractal dimension was calculated. Because in the surrogate data, the similarity of each signal pair has a downward trend, similarity measures, also, have to follow this trend.

### 2.6. Thresholding method

Presently, for studies on complex brain network, there is no consensus over selection of a specified threshold (Achard & Bullmore, 2007). Pursuant to studies already carried out, each element’s absolute value (positive and negative values were only contained in Pearson’s matrix) was applied as the inter-regional functional connectivity (Bassett et al., 2008; Braun et al., 2012; Wang et al., 2011; Zhang et al., 2011). Ultimately, we thresholded these matrices of adjacency into a binary matrix.

In this study, for the networks properties to be investigated, we employed a range of threshold values, from 0.1 to 0.5, with an increment of 0.1. In this method to derive adjacency matrices, we thresholded them in a proportional manner (i.e. normalization by “wiring cost” – e.g. 0.1 represents 10% of the strongest connections being maintained as links). For each individual, a connectivity matrix of 90×90 was obtained and the topological organization of functional networks of the whole brain analyzed in compliance with graph theory, taking each ROI into account as a node and the functional connectivity as an edge.

### 2.7. Topological properties of brain networks

Of all the multiple matrices of network we needed to measure for the assessment of the small-world properties (Sporns & Zwi, 2004), the *Clustering Coefficient* (CC) and *the mean minimum Path Length* (PL) are regarded to be the major components of the small-world network. The *clustering coefficient* ratio, 0<CC_i_<1, describes the range or amount of potential connections that the closest neighbors of a node actually has (Liu et al., 2009):

(7) $$\mathrm{CC}=\frac{1}{N}\sum_{j=1}^{N}\frac{\#E_{j}}{\#\frac{V_{j}(\#V_{j}-1)}{2}}$$

The total number of nodes of a network, the number of edges by which node j’s neighbors are connected to one another and the number of node j’s neighbors are illustrated by N, #E_j_ and #V_j_ in this equation, correspondingly. Moreover, we will have L (i.e. the minimum path length), dividing the shortest path lengths in average by each potential vertices pair:

(8) $$L=\frac{2}{N(N-1)}\sum_{S=1}^{N-1}\sum_{k=s+1}^{N}\min\{L_{i,j}\}$$

In the above formula, the path length is determined by the number of edges the path encompasses. Also delineates the shortest length (i.e. the i^th^ or the j^th^). The average minimum number of connections linking any two nodes of the network is referred to as the Characteristic Path Length (CPL). Furthermore, we computed, for a CC and CPL graph containing as many nodes as edges, the corresponding parameters, according to what and determine. These random networks were established to obliterate any structure of local adjacency, when the primary level of distribution is kept. This was carried out by each edge being reattached in the original network, 1000 times in average, randomly (Hayasaka & Laurienti, 2010; Newman, Strogatz, & Watts, 2001). Random networks require a shortest path length in average which is small, having limited local interconnections resulting in a small C_rand_ and L_rand_. The graph, on the ground that it has a small shortest length in average and its average *clustering coefficient* is strikingly greater than a constructed random graph on the same number of nodes, would be regarded as small-world. It has been demonstrated that, supporting effective parallel transmission of information at a comparatively low cost, economical small-world properties with high *global efficiency* (E_global_) are owned by the brain functional networks. The harmonic mean inverse of the least absolute path length between each nodes pair, is delineated as (Liu et al., 2008).

(9) $$E=\frac{1}{N(N-1)}\sum_{i\neq j\in G}\frac{1}{L_{i,j}}$$

For two groups of addicts and normal individuals, *clustering coefficient*, characteristic path length and efficiency were computed for edge definition using the three methods of LC, MI and BCFD, respectively. It should be noted that the nature of the graphs obtained by these three methods is different to some extent, where LC method quantifies the linear interactions between the nodes, MI calculates the shared information and BCFD computes the self-similarity. Thus, the values of *clustering coefficient*, path length and efficiency in the graphs obtained by LC and MI and BCFD methods will be different. But, regardless of the nature of these three graphs and the varied values of the features, the interpretation of *clustering coefficient*, path length and efficiency will be the same in all three graphs.

### 2.8. Statistical analysis

To evaluate the brain network properties, statistical difference between addict participants and normal controls, a test of non-parametric permutation was applied (He, Chen, & Evans, 2008). In the first place, we calculated the network topological properties inclusive of *Clustering Coefficients*, Characteristic Path Length (CPL) and *Global Efficiency* (GE). Next, each individual’s regional cortical thickness measures were assigned to either group randomly. In addition, we recomputed LC, MI and BCFD matrices for each of the 2 groups randomized and, for the network property measure, obtained a new value. We repeated this process of randomization 1000 times. Because less than 5 percentile of intergroup difference in the distribution of permutation is more than the discrepancy in the observed group, the significance would be reached. For each threshold, the procedure was repeated.

## 3. Result

Investigated from a functional integration viewpoint and in line with LC, MI and BCFD methods, methamphetamine addiction problems were considered through graph topological properties. In order for the network to be built, for both methamphetamine users and normal controls, graph theory was applied, enabling us to analyze the topological properties of functional brain networks between these two groups.

By generating the surrogate data, we aim at evaluating the performance of each method in several pairs of time series, before they are applied for the real data. That is because these methods are used as the similarity measure of two time series from two fMRI voxels. These similarity measures are compared and assessed using the time series with linear and non-linear behavior, about which we have prior knowledge. To evaluate LC, MI and BCFD methods, two surrogate data sets generated by the randomization process (as linear behavior) and by the logistic equation (as a non-linear behavior) were used.

**Figure 3.**
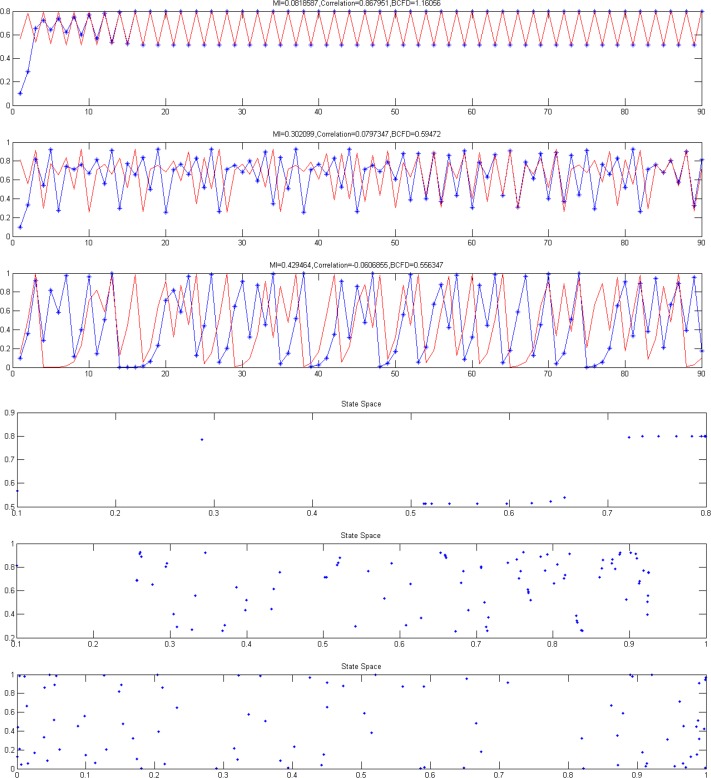
Six samples of time series created by logistic equation, by increasing the A, complexity of each pair has been increased.

Figure 3 shows the results of creating six time series using logistic equation. Upper signals correspond to less nonlinearity and more linearity and lower signals correspond to more nonlinearity and less linearity. As can be seen, there are a few similarities in the signals. We aim at quantifying these similarities using BCFD and evaluating the results of BCFD versus LC and MI.

As seen in Figure 3, with an increase in the value of A in logistic equation, complexity increases, interpreted based on an increase in the distribution of points in the phase space, while the similarity and connectivity of the two time series decrease in a non-linear manner. For these six typical time series (3 pairs), linear correlation, mutual information and box counting were executed and the similarity measures were computed. Table 2 displays the results.

**Table 2.** The results of computing LC, MI and BCFD for six samples of time series using Logistic equation.

	**LC**	**MI**	**BCFD**
Linear	0.8680	0.0818	1.1605
Relatively nonlinear	0.0797	0.3020	0.5974
Pure nonlinear	−0.0607	0.4294	0.5563

As seen in Table 2 and based on the visual comparison, by increasing nonlinearity, LC has no significant changes in the values of these three methods for nonlinear range and just has a good performance in linear range, MI has an increasing trend representing that mutual information functions well just in nonlinear range. Among these three algorithms only BCFD has significant changes and shows the capability of its discrimination for similarity measure in both linear and nonlinear ranges.

**Figure 4.**
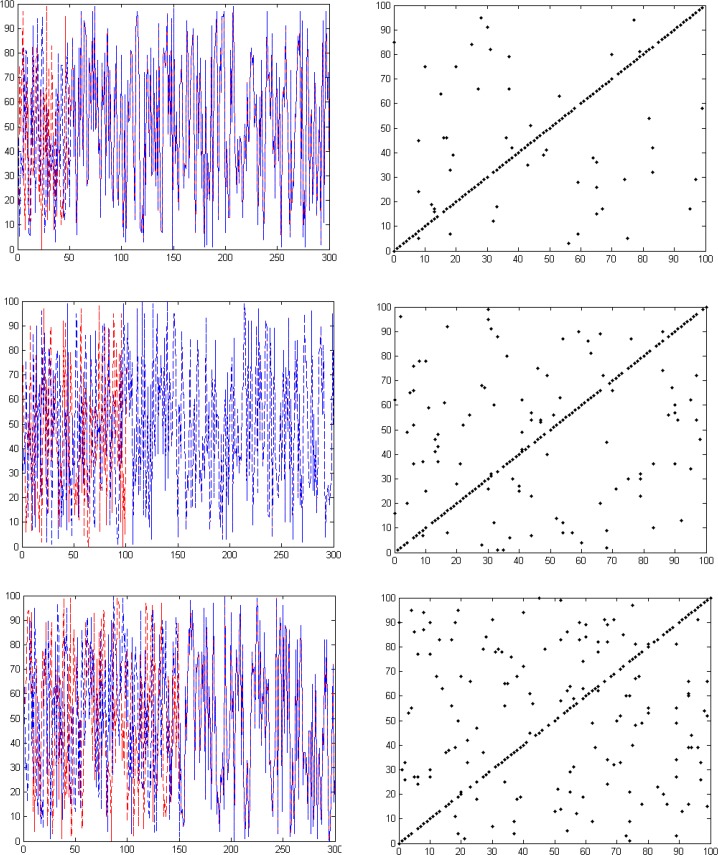
Six samples of time series created by randomization method, increase in the distribution of points in the phase space lead to less simialarity of two time series.

Figure 4 shows the results of creating six time series using randomization method and corresponding phase spaces. Upper signals correspond to more similarity. By reducing the time series similarity, dispersion of points in phase space has been increased.

Based on our statistical analysis using BCFD and consistent with several previously conducted studies on addiction, there was a significant difference between groups in *global efficiency*, *clustering coefficient*, as well as Characteristic Path Length (CPL) at different thresholds (Ersche et al., 2006; Jiang et al., 2013).

**Figure 5.**
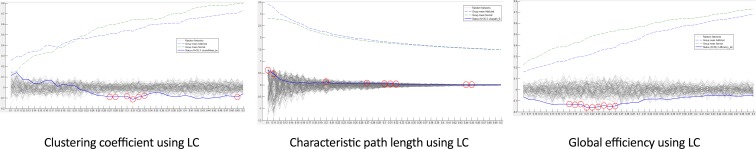
The results of statistical analysis for intergroup differences using LC (red circles show significances at P<0.05).

Figure 5 depicts CC, CPL and GE schemes of the whole-brain functional network changing with the thresholds in both addict participants and normal controls and obtained with the application of LC, MI and BCFD methods. As can be seen in Figure 5a, Figure 5b and Figure 5c, for LC method, significant differences between groups in CC, CPL and GE were observed. As desired and compared to the random networks, relatively higher CC and GE, except CPL, were exhibited by the networks of addict and normal participants. Thus at the red circle thresholds, there were differences in CC, CPL and GE considered significant (red circles, P<0.05). Only CC and GE of normal subjects valued higher.

**Figure 6.**
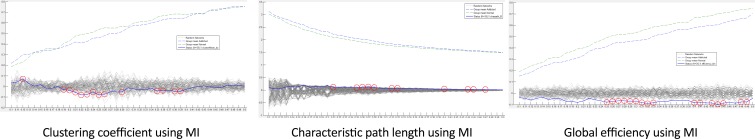
The result of statistical analysis for intergroup differences using MI (red circles show significances at P<0.05).

Graph topological properties of both addict subjects and normal controls using MI method is presented in Figure 6a, Figure 6b and Figure 6c. In this respect, similar to the analysis using LC, significant differences at a number of network thresholds for CC, CPL and GE (red circles, P<0.05) were revealed. Conforming to the results, as compared to those of addict subjects, relatively higher CC and GE were exhibited by the networks of normal controls. Furthermore, implying that MI is more robust than LC to extract to the differences in graph topological features, the number of significant differences using MI is more than those through LC method.

**Figure 7.**
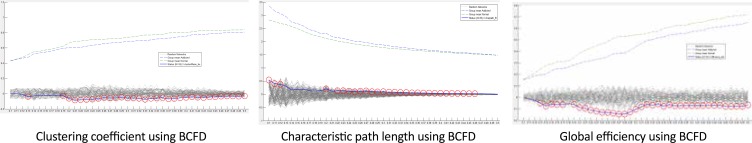
The result of statistical analysis for intergroup differences using BCFD (red circle show significances at P<0.05).

Figure 7 illustrates the statistical analysis of the graph topological properties using BCFD. Once more, identical to the analysis utilizing LC and MI, as compared with normal controls, brain networks of addicted participants display significant differences in CC, CPL and GE. In this method, the number of significant points is observed at a wide range. Thus, BFCD holds more homogenous significant variations insinuating that, compared with normal controls, the differences in addicts’ brain networks in almost all network thresholds can be differentiated by BCFD method and, besides, represent greater validity and reliability of the brain network analysis using graph theory. According to the results, in all circumstances, all three topological features in healthy individuals are more than in addicts, with the exception of CPL, by whom the main hypothesis of this study is asserted to be vindicated.

## 4. Discussion

A prominent approach to the analysis of fMRI data, in particular the resting state data is Functional Connectivity (FC). Two common methods of functional connectivity analysis are graph theory and seed-based analysis which employ Linear Correlation (LC) measures. An implicit assumption of Gaussianity is applied to the dependence structure by utilizing linear correlation measures (Chang & Glover, 2010). It is of a great importance to bear in mind that non-Gaussianity may result in the false detection of nonstationarity. It should also be kept in mind that, rather than with neuronal activations, we are dealing with the level of fMRI BOLD signal; the non-neural sources of variation are likely to influence, to some extent, the contributions of both the Gaussian and non-Gaussian. Due to generality, a specific position is held by Mutual Information (MI) among a great number of possible nonlinear FC measure candidates. Theoretically, adopting an arbitrary form of dependence association among the variables, without any apriority model limitations on its form, would be general enough. MI features provide us with the opportunity not only to examine the appropriateness of linear correlation for fMRI time series, but also to quantify the information disregarded using linear correlation. Owing to greater sensitivity of MI to higher order statistics considering only the second order (Smith et al., 2011), as compared to LC, an extra amount of information is resulted and the possible contribution of non-linear alternatives over the Pearson correlation coefficient is bounded.

Currently by employing the elements of chaos theory, modern neuroscientists have been able to recognize the fractal properties present in the brain functions (Expert et al., 2011; Papo et al., 2014; You & Stadler, 2012). However, until just the recent time, there was no analytical method for the objective explanation of the brain complexity. However, the question of which interdependence or connectivity measures to be applied is not generally responded. The crucial trade-off factors would probably depend upon a specific dataset and the regarding scientific problem (Hartman et al., 2011).

**Figure 8.**
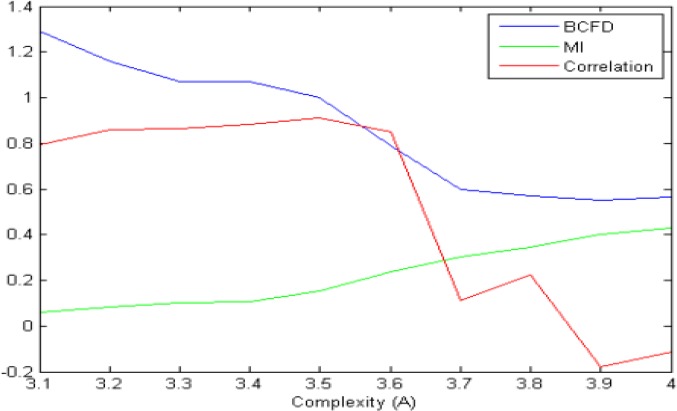
General trend of LC, MI and BCFD with increasing complexity.

The surrogate data analysis conducted applying the logistic equation may give us the insight to fractal behavior impact on the brain complex networks. Figure 8 displays the performance of these 3 algorithms with increasing the complexity. As desired, correlation has a good performance in only linear range because of its fluctuation. MI has a good performance in only nonlinear, but BCFD performs well in both linear and nonlinear ranges, expressing that BFCD is a better dependency measure for comparing time series to define the edge of graph for graph-centered network analysis of the brain.

**Figure 9.**
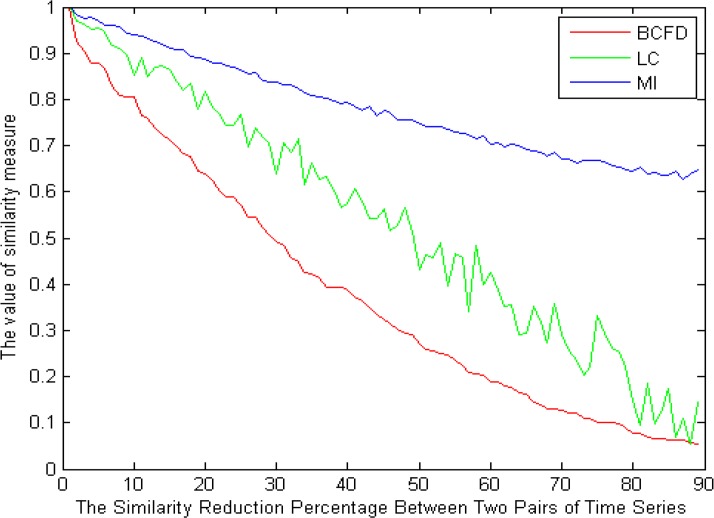
The comparison of the methods used for obtaining similarity measure by a decrease in the value of similarity resulted from the randomization method.

In order to better evaluate the similarity measures applied in the brain functional connectivity using the graph theory, other surrogate data were created using the randomization process. In these data, as shown in Figure 9, in the first step where the two signals are completely similar to one another, their values have to be the maximum value. Since in establishing the random signal pairs, the trend is a monotonic downward trend, the values of similarity measures have to lack fluctuations. The last important point is that, in the last stage, the similarity of the pair of signals would be a specific number; hence, in the last phase, similarity measures should have a value similar to that specific value.

As seen in Figure 9, all the three similarity measures are started from the maximum value, which seems reasonable, knowing that two signal pairs are completely similar. However, compared to the other methods, the Linear Correlation (LC) has more fluctuations, which is not desirable due to less similarity of each signal pair in proportion to the prior signal pair in the surrogate data.

In spite of its lower fluctuations, Mutual Information (MI) method does not perform greatly for low similarity values. That is because, in the last stages where the specific value of 30% in the surrogate data, for example, has been already given to the similarity, a much higher value is shown by the use of this measure. Among the similarity measures, Box-Counting Fractal Dimension (BCFD) seems to have been able to follow all the existent behavior, and thus, it can have a better performance for the graph edge determination in brain functional connectivity analyses.

By reducing the similarity value, LC method has a great number of fluctuations and MI follows the variations in the similarity reduction with a very little slope, whereas FD follows the variations in the similarity reduction well. Being repeated 1000 times, the results from the statistical analysis showed no significant difference between LC, MI and FD methods in the data generated through randomization. Similar to the randomization method, we reduce the similarity of the time series to one another in a linear manner, such a conclusion seems reasonable. That is because, in fact, the data still have a linear relationship with each other. However, in the data created by the logistic equation, a significant difference was observed between the three methods. Similarly, this result is not far-fetched, as with an increase in the value of A in the logistic equation, complexity (distribution of points in the phase space), i.e. the non-linear interaction between the time series will increase. Regarding the evidence cited in the surrogate data, it can be concluded that FD seems to be a more appropriate criterion for the graph edge determination in functional connectivity studies.

In addition, it is proven that the brain, as a complex structure, is fractal (Papo et al., 2014). To express the brain complexity and present an index for it, the complexity of the time series of fMRI voxels can be investigated between the test group and the control group. An expression of complexity introduced in this study was the point’s distribution of the time series in the phase space, a kind of deviation from the linearity and nonlinearity of the interactions between the brain regions of addicts, compared with normal controls, quantified and evaluated using BCFD method. The use of BCFD method for quantifying the complexity of the interactions of fMRI time series and thus, providing a criterion for the brain complexity is a benefit not achievable through the conventional methods of LC and MI. However, applying other concepts to interpret and assess the complexity of the brain requires further studies.

There is a number of well-established circuits characterizing the cognitive impairments of addicted brain; namely decision-making, impulsivity, attention disturbances and assigning emotional valence circuits (Lee & Pau, 2002; Lee et al., 2005; Li & Sinha, 2008; Volkow, Fowler, & Wang, 2003). Studies have also suggested some irregularities in these circuits for methamphetamine dependents (Monterosso, Aron, Cordova, Xu, & London, 2005). To this end, studying these circuits in MDI through a graph theoretical perspective is beneficial. In the current study, alterations were found in MDIs universal parameters of the brain functional networks, in comparison with those of NCs. Statistical analysis revealed a significant decrease in *clustering coefficient*, *global efficiency* and small-world indices and increase in characteristic path length of MDIs.

One of the indices characterizing the manner in which the brain networks are shifted to either a regular or a random network is the *clustering coefficient* (He & Evans, 2010). Studies have previously shown a decreased *clustering coefficient* in ADHD individuals justifying their attention disorders (Wang, Li, Metzak, He, & Woodward, 2010), this may also be the case for our MDIs as attention deficit is a commonly reported cognitive impairment in meth addiction (Sim et al., 2001).

Universal processing and transfer of information from each brain region to all the other ones is taken into consideration by *global efficiency*, which, to ensure effective interactions or rapid information transfer over the distant cortical regions involved in the basis of a great number of cognitive processes, predominantly relates the long-range connections (Latora & Marchiori, 2001). Low *global efficiency* is linked to lower IQ score (Li et al., 2009; Van den Heuvel, Stam, Kahn, & Pol, 2009), which may be due to a slower information process, a critical subscale in IQ score; consistently lower *global efficiency* can be indicative of decreased information process speed in MDIs.

The mean minimum number of links connecting any two nodes within the network is considered as the characteristic path length of a network. Giving an overview of information transfer effectiveness and measuring the functional integration of the whole brain, the capability for the brain network parallel information propagation is quantified by the characteristic path length of a network (Tschernegg et al., 2013). Obviously, a longer characteristic path length will make for a more time consuming and/or less efficient integration of modules. Increase in characteristic path length in our participants can be a reason for the observed psychomotor retardation of brain functions in abstinent psycho stimulant-dependent individuals (Pulvirenti & Koob, 1993).

**Figure 10.**
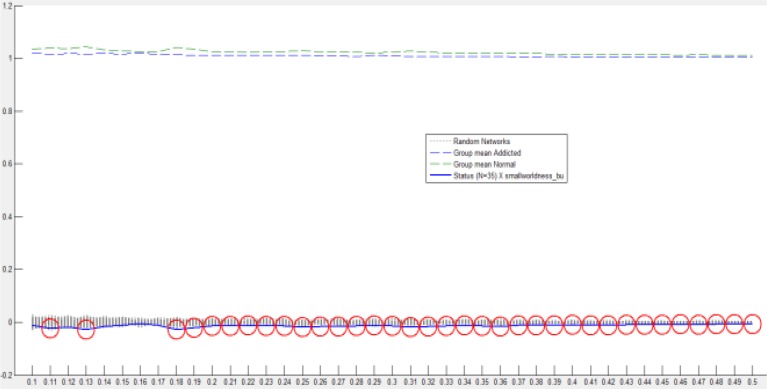
Small-worldness of MDIs and NCs using BCFD.

The model of small-world network topology is considered by a high local *clustering coefficient* and shortest path length (Rubinov & Sporns, 2010). Small-world properties lead to maintaining highly effective, specialized modular information process and fast global information transfer in a network (Kaiser & Hilgetag, 2006). Similar to earlier studies on functional networks of the human brain (Liao et al., 2010; Liu et al., 2008; Supekar et al., 2008; Tian, Wang, Yan, & He, 2011; Zhang et al., 2011), in this study, the small-world properties of MDIs’ and NCs’ resting networks were investigated over a range of threshold values. Statistical comparison of the two groups showed that small-world properties were conserved by both MDIs’ and NCs’ whole-brain functional network. The small-world scalar was smaller in MDIs than the NCs (Figure 10). Small-word property increases through brain development, representing a shift of brain networks towards regularity (Park et al., 2015); indicating improved decision making in adults. Inversely, low small-world scalar in our study may be a reason for common cognitive impairments in MDIs, especially disrupted decision making (Kohno, Morales, Ghahremani, Hellemann, & London, 2014) and impulsive behaviors, a hint of a shift towards randomness.

By comparing the edge definition methods and the features derived from functional connectivity graph, it is concluded that there is a significant difference in the brain network topological features of the addicts, compared to those of the control group; in all three edge definition methods, the difference was obtained in terms of network topological features. The number of significant points, however, are more in BCFD method than in LC and even MI. As stated in Liu et al. study (Liu et al., 2009), the greater number of significant points, regardless of the connectivity threshold, can be a criterion for the comparison of these methods. These materials can certify the proof of the main and sub-hypotheses of this research.

## 5. Conclusion

According to the gathered body of evidence, a more powerful manner to appreciate the brain networks topological principles is put up by the graph theoretical analysis of neuroimaging data applying BCFD than through LC and MI methods. Using resting-state fMRI and a graph theory method, we aimed at investigating the functional network of the whole-brain in MDIs. As lower *clustering coefficient*, *global efficiency* and lessened small-worldness indicate, the whole-brain functional networks in MDIs, compared to those in normal controls, were pointed out to be probably shifted toward random organization.

On the whole, the present study found disruptions in the whole-brain functional networks topological graph properties of MDIs. The findings are valuable to better understand the underlying mechanisms of addiction. Due to such efforts, recently developed research areas of interest to all basic scientists and clinical researchers are being opened up into organizational mechanisms of the brain. Considering the features of graph theory in establishment of network alterations, it seems to be a suitable biomarker for monitoring addiction treatment and detecting the treatment progress ahead of significant clinical symptoms.
